# Short Glass Fiber Modifier Trisilanol–Isobutyl Polyhedral Silsesquioxane as Interfacial in Polypropylene Matrix: Effect of Flame Retardation and Mechanical Properties

**DOI:** 10.3390/polym16162235

**Published:** 2024-08-06

**Authors:** Ana Beatriz Morales Cepeda, Diego Armando Quiñones Lopez, Saúl Sánchez Valdez, Luis E. Cabrales Arriaga, David Victoria Valenzuela, Hernan Peraza Vazquez

**Affiliations:** 1 Centro de Investigación en Petroquímica, Tecnológico Nacional de México/Instituto Tecnológico de Ciudad Madero, Parque Industrial Tecnia, Bahía de Aldahir S/N, Altamira C.P. 89600, Tamaulipas, Mexico; diegoarmandoquinones@gmail.com (D.A.Q.L.); david.vv@cdmadero.tecnm.mx (D.V.V.); 2 Department of Polymer Processing, Centro de Investigación en Química Aplicada, Saltillo C.P. 25294, Coahuila, Mexico; saul.sanchez@ciqa.edu.mx; 3 Department Mechatronics Engineering, California State University, Monterey Bay, Long Beach, CA 90840, USA; lucabrales@csumb.edu; 4 Centro de Investigación en Ciencia Aplicada y Tecnología Avanzada (CICATA), Unidad Altamira, Instituto Politécnico Nacional, Mexico City C.P. 89120, Tamaulipas, Mexico; hperaza@ipn.mx

**Keywords:** polypropylene, glass fiber, TSI-POSS, mechanical properties, flame retardation

## Abstract

In the present work, short glass fiber is superficially modified with different concentrations of polyhedral oligomeric silsesquioxanes (Trisilanol–Isobutyl, TSI-POSS) for processing as a filler in a polypropylene matrix (PP). It is observed that increasing the amount of TSI-POSS increases the fracture point and tensile strength; the opposite is the case for the strength impact property. The behaviors of both dynamic mechanical and thermal analyses are also observed. The flame behavior, i.e., the burning rate, decreases with increasing TSI-POSS in the polymers.

## 1. Introduction

Glass fiber and polypropylene (PP) composites are widely used in the automotive industry due to their low costs compared with other polymeric materials, such as polyamides and PBT. However, because PP is a nonpolar and inert thermoplastic, its interaction with other polar materials, such as glass fiber, is complicated. To achieve this interaction, coupling agents that promote adhesion between the matrix and the reinforcement material are sought.

In recent years, the use of oligomeric polyhedral silsesquioxanes (POSSs) has been widely studied to modify the surface of various types of materials [1,2,3,4,5,6], as they are effective agents that can be incorporated into polymeric matrices via graft copolymerization and even through traditional mixing processing methods.

The incorporation of these materials by themselves improves various physical and mechanical properties of the material in which they are dispersed. However, they can act as an interface in the case of composite materials, as it has been observed that they create covalent bonds; the functional group “OH” has been found on the surface of many materials, and the alkyl groups that are also present in the structure of silsesquioxanes provide compatibility with polymeric resin [7].

Other authors have studied the interaction of POSSs with other polymers, such as polymethylmethacrylate and elastomers. POSSs have improved mechanical properties and flame stabilities. They find applications in aerospace and with polymers exposed to high temperatures [6].

In this study, the surfaces of short glass fibers were modified with TSI-POSS to obtain composite materials using polypropylene as a matrix. The incorporation of TSI-POSS into polypropylene composites and the modification of glass fibers with TSI-POSS, both in small amounts, resulted in thermomechanical properties slightly superior to those of polypropylene with fibers. Additionally, flame-retardant properties were found in polypropylene composites with TSI-POSS-modified glass fibers. The scientific literature on the modification of short glass fibers with siloxanes to improve the properties of polyolefins was reviewed, but no related research was found.

This research proposes the possible use of polypropylene composites with TSI-POSS-modified glass fibers in high-intensity applications.

## 2. Materials and Methods

Trisilanol–Isobutyl (TSI-POSS) (C_28_H_66_O_12_Si_7_), with a molecular weight of 791.42 g/mol and thermal stability at 210 °C, is a hybrid molecule with an inorganic silsesquioxane in the nucleus, isobutyl organic groups linked at the vertices of the structure in the form of a molecule box, and three active silanol functional groups; it was acquired from the Hybrid Plastics company (Hattiesburg, MS, USA). The polypropylene (PP) used was Profax 6331 from the company Indelpro S.A. de C.V. (Altamira, Mexico), with a Melt Index (MFR) of 12 dg/min (ASTM D1238 [8]). Fermont brand n-hexane was used as a solvent for the TSI-POSS. The glass fiber used was Cratec 497A-14C from the Owens Corning company (Ciudad de Mexico, Mexico); it was cut to 4 mm in length and 14 µm in diameter. Its mechanical properties were as follows: tensile strength, 3100–3800 MPa (ASTM D2101 [9]); elongation, 4.5–4.9% (ASTM D2101); elastic moduli, 11.0–118 MPsi (Sonic Modulus); and creep, none. The physical properties of the glass fiber gauge included a specific gravity of 2.52–2.68 (ASTM D1505 [10]); it is designed to be used as a reinforcement material in high-temperature thermoplastic polymers.

**Figure 1:**
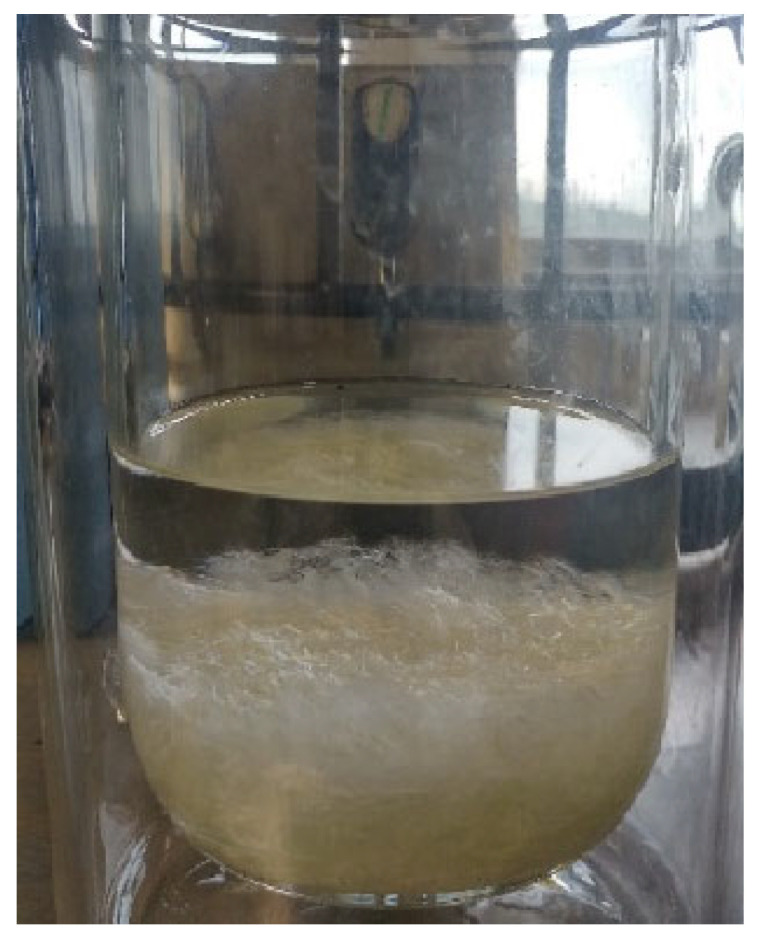
Glass reactor used during surface modification of glass fiber.

For the modification of the glass fiber surface, TSI-POSS (Table 1) was stirred in 400 mL of hexane until it was completely dissolved. Afterwards, the glass fiber was added. Each sample was allowed to stand for 24 h at room temperature (Figure 1). After this time, each sample was stirred at 230–250 rpm at room temperature for 2.5 h. The stirrer was removed from the mixture, which was left to rest in a crystallizer at room temperature for 24 h. Later, the modified fiber was placed in a rotary evaporator to eliminate the excess hexane [11,12,13].

A twin-screw extruder was processed in a Thermo Scientific TSE 24-MC double extruder with a C1 Termolita 2014 (Waltham, MA, USA) profile; the feeder was from the Brabender Tech brand (Duisburg, Germany), and it had an L/D ratio of 40:1 [14]. The equipment was programmed to operate at a speed of 100 rpm, with a flat temperature profile of 200 °C in the 10 heating zones of the extruder barrel. The feed was 1%, and temperatures between 212 and 228 °C were reached at 30 to 35 bar pressures. Table 1 shows the composition of the samples used in this investigation.

**Figure 2:**
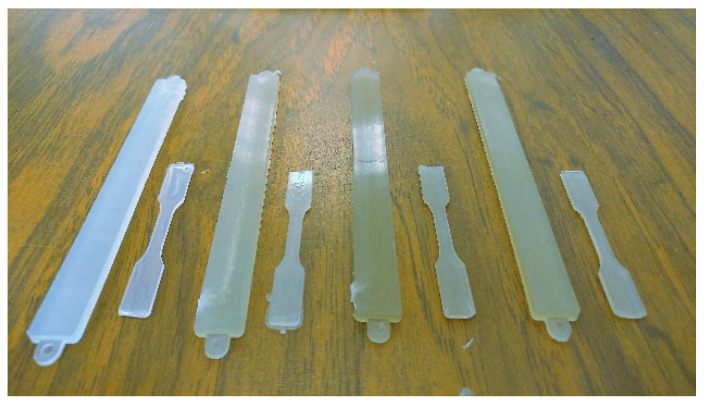
Specimens were obtained using injection extrusion for the tests.

Specimens were developed for a DMA analysis—as well as tension and elongation tests—as shown in Figure 2, according to the ASTM D638 standard [15].

For this process, a Trubor Co. (West Boylston, MA, USA) model 20JH-RS-1 injector was used; the configuration of the two heating zones of the equipment barrel was 235 and 240 °C. The procedure that was carried out is typical for this type of process, whereby the material was loaded into the barrel, the polymer was injected into the metal plates with the desired shapes, the material was cooled, and specimens were obtained. Between 8 and 12 specimens of two types were obtained for each sample of the composite material. The first type corresponded to the tensile tests, and it was called “dog bone”; measurements were conducted according to the ASTM D638 standard. The second type was in the form of plates with measurements of 125 × 12.7 × 3.2 mm, which were cut to 64 × 12.7 × 3.2 mm; a measurement was taken corresponding to the ISO-180 standard [16] for Izod impact tests with notches, and another one was taken at 45 × 12.7 × 3.2 mm for a DMA analysis.

The dynamic mechanical analyses were carried out using a DMA Q800 from TA Instruments (New Castle, Delaware, USA) with a multi-frequency module at 1 Hz and a dual cantilever jaw. The equipment was configured to maintain a heating rate of 5 °C/min between 40 and 150 °C. The samples used had a rectangular geometry of 40 mm long, 12.7 mm wide, and 3.2 mm thick.

Regarding the mechanical properties, tension tests were carried out using an Instron Model 5969 Materials Testing System machine (Norwood, MA, USA) with a capacity of 50 KN. Blue Hill software (https://www.instron.com/en/products/materials-testing-software/bluehill-universal, (accessed on 27 July 2024)) was used to manipulate the equipment and collect data. The dog bone-shaped specimens that were standardized using the ISO 527 standard [17], type 5-A, were used. The operating conditions were 30 mm/min at a temperature of 30 °C, with three repetitions per sample. Izod impact strengths were measured following the ASTM D256 method [18].

Fourier transform infrared spectroscopy: Infrared spectroscopy analyses were carried out on a Perkin Elmer Spectrum One (Shelton, CT, USA), with a diamond-tip ATR ((Shelton, CT, USA). The original and post-treatment glass fiber samples and films of the composite material samples were analyzed. The equipment was adjusted for 12 scans for each sample, and the resolution was 4 cm^−1^.

X-ray diffraction: Analyses were carried out on a PANalytical Empyrean X-ray diffractometer (Worcestershire, United Kingdom). The specimens used in the mechanical tests were used; the interval was from 2.99 to 55° in 2θ. The X-ray tube was made of copper (CuKα with λ = 0.154 nm), with a voltage generator of 45 kV and a current of 40 mA. Analyses were carried out per sample, and the average of the intensities recorded was calculated for later graphing. The data were processed and analyzed using OriginPro 2023 software.

A UL-94 horizontal burning test was performed according to ASTM D635 [19] using a FTT Dual Cone Calorimeter (East Grinsted, West Sussex, United Kingdom) under a heat flux of 35 kW/m^2^, using three specimens with an area of 100 cm^2^ to obtain the average values. Conventional data (the time of ignition, peak heat release rate, and total heat evolved) were determined.

A light microscope, the Zeiss brand AXIO Lab 10 model, (Oberkoche, Germany), was used; a 10× lens, Motic software Image 3 Plus, and a Moticam 1SP1.3MP camera (Xiamen, China) were also utilized.

The samples were analyzed under a nitrogen atmosphere using a TA instrument DSC/TGA model SDT Q600 from New Castle, DE, USA.

## 3. Results

For the analysis of the glass fiber on PP, the average fiber length was studied and observed using an optical microscope with film samples. Table 2 shows that the longest fibers were those treated with 0.5% TSI-POSS; however, in general, these fibers were considerably reduced in length from the original, probably due to the design of the equipment (Termolita 2014). The samples were also extruded twice to achieve a better fiber dispersion, as it was impossible to achieve a uniform feed in the extrusion due to the glass fiber’s characteristics.

Fu et al. [20] observed a similar behavior, with the length decreasing after the injection process from 3 mm to 0.93 mm and from 15 mm to 1.14 mm.

### 3.1. FTIR Analysis

**Figure 3:**
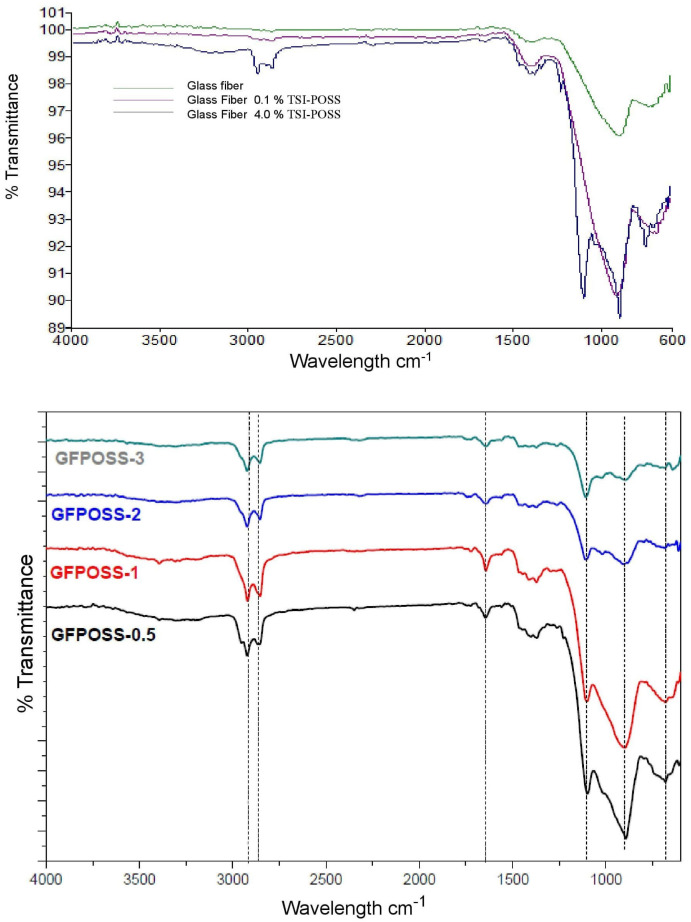
FTIR spectra of glass fiber modification using TSI-POSS.

Figure 3 shows a summary of the samples that were treated with TSI-POSS. Two peaks can be observed in the region between 2950 and 2850 cm^−1^ (2925 and 2862 cm^−1^), and they become more prominent with the increasing concentration of TSI-POSS in each sample. These peaks are typical of C-H stretches, directly related to the isobutyl (CH_3_)_2_-CH-CH_2_-) constituents in the TSI-POSS, and they were not initially found in the glass fiber sample without any modification. Likewise, a thick band appears between 1468 and 1370 cm^−1^, corresponding to the bending/deformation of the functional groups CH_2_ and CH_3_, both present in the isobutyl molecular chain. Another wide and high-intensity peak can be observed in the region of 890 cm^−1^; the literature indicates that, between 1106 and 950 cm^−1^, the compounds made up of Si-OH form thick peaks; this type of signal has been previously described [2,12] and denotes the condensation reaction between the silanol groups (Si-O-Si) with the OHs on the surface of the glass fiber. All these signals indicate the adherence of the TSI-POSS in the glass fiber; the samples with 0.5 and 4% had more pronounced signals.

### 3.2. Dynamic Mechanical Analysis

**Figure 4:**
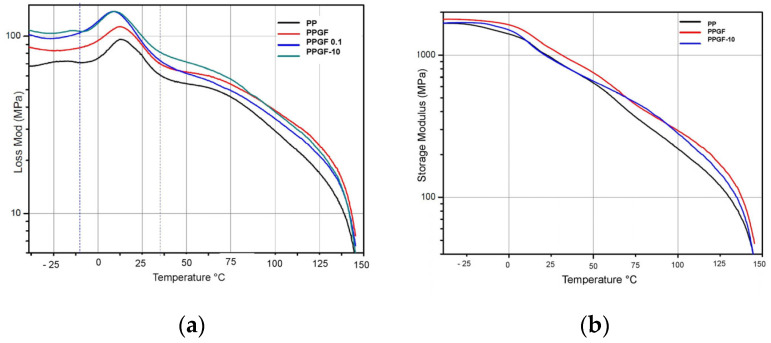
DMA analysis of (**a**) the loss modulus and (**b**) the storage modulus of PP-B, PPGF-B, and PPGF-10.

Figure 4 shows the DMA analysis of the loss and storage modulus of the PPGF, PPGF 0.1, and PPGF 10 samples. Table 3 shows that the pure PP had an E′ of 1658.69 MPa; the storage modulus of the sample with glass fiber without treatment increased by 7.02%, while the storage modulus of the sample with PPGF-10 returned to almost the same as that of the pure PP, at 1659 MPa [21]. At the same temperature, the PPGF-4 sample registered a higher E′ of 1876.83 MPa; thus, its storage modulus increased by 11.62%. The PPGF-2 sample (treated with 2%) presented a decrease in the storage modulus of 57.9% with respect to that of the composite material sample without surface modification (PPGF-B), registering at 750.97 MPa. The PP first showed a drop in the storage modulus at −29.65 °C and another between 11 and 50 °C; both are related to second- and first-order transitions. The first drop did not appear in the curves of the composite material samples, except for in the samples with 3 and 10% TSI-POSS; this change was presumed to be a beta transition (T_β_) and is due to the movements of localized functional groups or the secondary chains of molecules because the free volume within the molecular structure of the material increases as it expands with temperature. According to several authors [22], T_β_ is related to the mechanical properties of materials, as it corresponds to the relaxation of polypropylene molecules in the amorphous section of the polymer [23]. The second drop in the E′ curves is due to a first-degree transition or α transition, also known as T_α_, T_g_, or glass transition. This is due to large laminar movements in the main chain of the polymer in its amorphous phase [24]. In the figures, this can be seen as a shoulder that is slightly more pronounced than T_β_, where the PP presents a T_g_ of 6.76 °C. The glass transition temperature of the PPGF-B sample of composite material decreased to 2.49 °C. As the TSI-POSS concentration increased, T_g_ increased slightly. Most authors and studies take more precise data to measure T_g_ at the maximum value in the most pronounced peak of tan δ, known as the maximum tan δ or damping factor; these graphs are shown later.

The analyses yielded curves with a broad peak ranging from −10 °C to 30 °C, indicating the transition between the glassy state and the rubber phase of the polymer. The virgin PP sample presented an E″ of 95 MPa at a temperature $T_{\alpha }^{E^{\prime \prime }}$ of 13.28 °C; at that temperature, the maximum heat dissipation per unit of deformation occurred. The blank sample of the composite material (PPGF-B) presented E″ and $T_{\alpha }^{E^{\prime \prime }}$ values of 113.76 MPa and 13.1 °C, respectively. Above the region of the phase transition, E″ suddenly decreased, indicating a considerable reduction in the viscosity of the material. In general, no clear trend was observed in terms of the loss modulus and the concentration of TSI-POSS; however, the maximum value of E″ did correspond to the sample with the highest concentration of PPGF-10, presenting as 137.93 MPa at 10.26 °C. All values are tabulated in Table 3.

**Figure 5:**
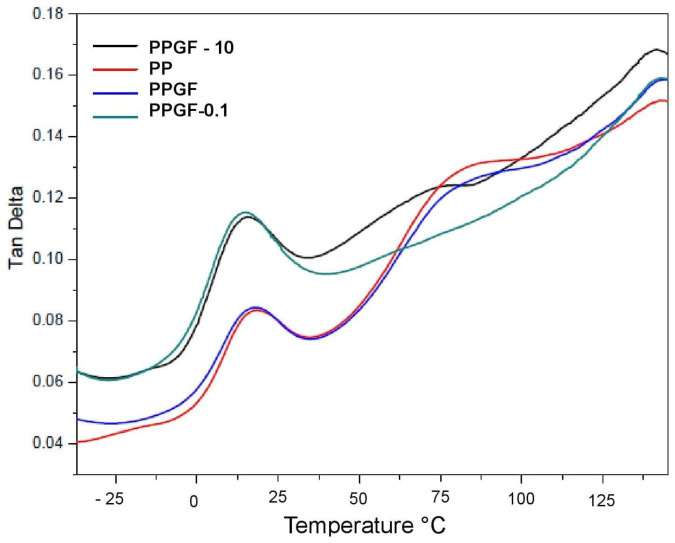
Loss factor or tan δ at 1 Hz from −40 to 150 °C.

This analysis included the results in Table 4 and Figure 5, from which it was easier to determine the T_g_ of the material. The virgin PP sample registered a T_g_ of 17.73 °C, with a maximum tan δ of 0.0833. The glass transition temperature of the PPGF-B sample effectively did not change, as it registered at 17.89 °C, with a similar tan δ of 0.0847. The samples with 0.1 and 10% of TSI-POSS showed a decreased T_g_: the former at 14.59 °C, and the latter at 15.52 °C; however, both had an increased tan δ. The lowest glass transition temperature was observed in the PPGF-0.1 sample at 14.59 °C. In general, tan δ increased with the incorporation of TSI-POSS, although a directly proportional behavior linked to its concentration was not observed. The above indicates that, contrary to what was expected, the adhesion between the matrix and the fiber decreased when incorporating TSI-POSS, as tan δ decreases as adhesion at the interface improves. Between T_g_ and the melting temperature (T_m_) are the transitions T_α_*, which are shown as a second peak with a lower intensity than that of T_g_; this indicates the temperature at which crystal–crystal slides are carried out in semi-crystalline polymers.

### 3.3. Mechanical Properties

**Figure 6:**
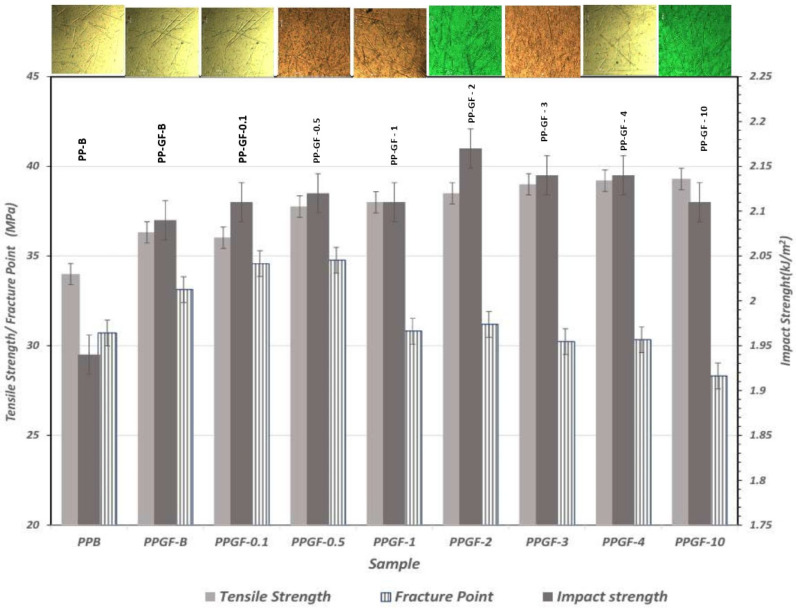
Results of tensile strength, fracture point, and impact strength and images of polymers.

The mechanical property results show that the increase in glass fiber content in the samples increased the tensile strength by 6.38%; however, in the composite samples with the highest TSI-POSS concentrations (Figure 6), the sample with 0.5% TSI-POSS added obtained a tensile stress that was 3.81% higher than that of the composite material sample without modification and 9.98% higher than that of the pure PP.

The PPGF-0.5 sample showed a tensile strength of 37.76 MPa at an elongation of 9.63%; the samples with a higher concentration of TSI-POSS showed an increase in this property. The PPGF-10 sample showed a tensile strength increase of 10% compared with the pure PP, and 13.54% compared with the PPGF-B sample. Regarding the fracture point, the PPGF-0.5 material registered the greatest force necessary to reach said point, registering at 34.77 MPa at 14.84% elongation, demonstrating increases of 11.67% and 4.71% with respect to those of the pure PP [25,26,27,28]. The sample that required less force to reach the breaking point again had a 10% concentration of TSI-POSS, decreasing by 14.54% compared with the unmodified composite sample; this is due to the effect of anisotropy when more fiber is modified during processing in a twin screw. The Termolite 2014 extruder configuration allows for mixing the glass fibers within the polymer matrix. As shown in Figure 5, the higher the concentration of TSI-POSS in the glass fiber, the more it is observed in the matrix (see polarized light photographs), and its notched impact strength and tensile strength are slightly higher. Adding short glass fibers to recycled polypropylene has been shown to increase the impact of notched strength on pure polypropylene [27,28,29]. This suggests that incorporating short glass fibers can alter material properties, which may have implications for flame retardancy and mechanical performance [13,21,27,30,31].

### 3.4. X-ray Diffraction

**Figure 7:**
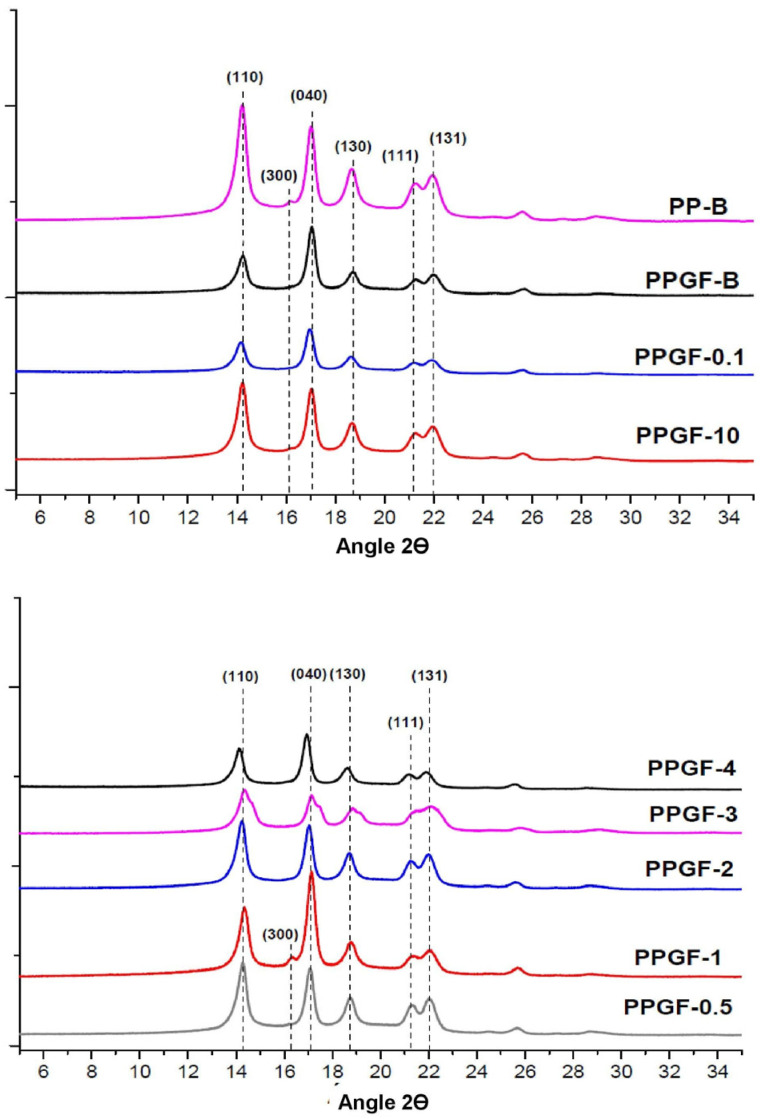
X-ray diffraction patterns.

It has been reported that the characteristic diffraction patterns of PP correspond to the lattice planes (110), (040), (130), (111), and (131) in the 2θ angular positions of 14.2°, 17.1°, 18.6°, 21.2°, and 21.9°, respectively, when it comes to the α (monoclinic) crystallographic phase, and that they correspond to (300) in the 2θ position of 16.3° when it comes to the β (hexagonal) phase [13]. Figure 7 shows the diffractograms obtained from the analyzed samples.

Table 5 shows the specific results of the 2θ positions of each diffraction response of the lattice planes and the interplanar distance between them, calculated using Bragg’s Law.

As mentioned above, PP is a polymorphic polymer that generally has two crystalline phases in its structure: a monoclinic phase, called the α phase, and a hexagonal phase, called the β phase. The former is predominant under normal processing conditions. The crystallographic unit β becomes more evident when PP is exposed to a high-temperature gradient or processed with a higher shear. In addition, it has been reported that when materials of a mineral or organic nature are added, the structure of PP becomes purely of the β type [27]; this type of phase is unstable and usually converts to the monoclinic type when the polymer is exposed to stretching through a melting or recrystallization process. Figure 7 shows that there was no significant change in the position of the reflections between all the samples. It was also observed that the intensity of the peaks decreased when adding the glass fiber compared with the pure PP sample. Zhu et al. [21], in a similar study where they used organofunctional silanes and a PP-g-MAH copolymer as surface modifiers of glass fiber, concluded that this phenomenon occurs because glass fiber acts as an anti-nucleating agent and that, when added to the PP matrix, the number of nuclei increases, while the size of the crystals decreases. This can be corroborated in Table 6, where the intensities recorded for each representative peak of the materials and the size of the iPP crystals are presented. Table 7 shows the results for response intensity and crystal size in radians using the Gaussian method and the Scherrer equation.

Another point to consider is that in the pure PP sample, a slight response was observed in position 16.2° in 2θ; this signal, corresponding to the plane (300), indicates that in the polymer used initially, there was a small portion of β crystals, but when the glass fiber was added, they disappeared. This is because, as is well known, the hexagonal phase of PP is very unstable and tends to transform to the α type when using nucleating agents [21], such as glass fiber.

**Figure 8:**
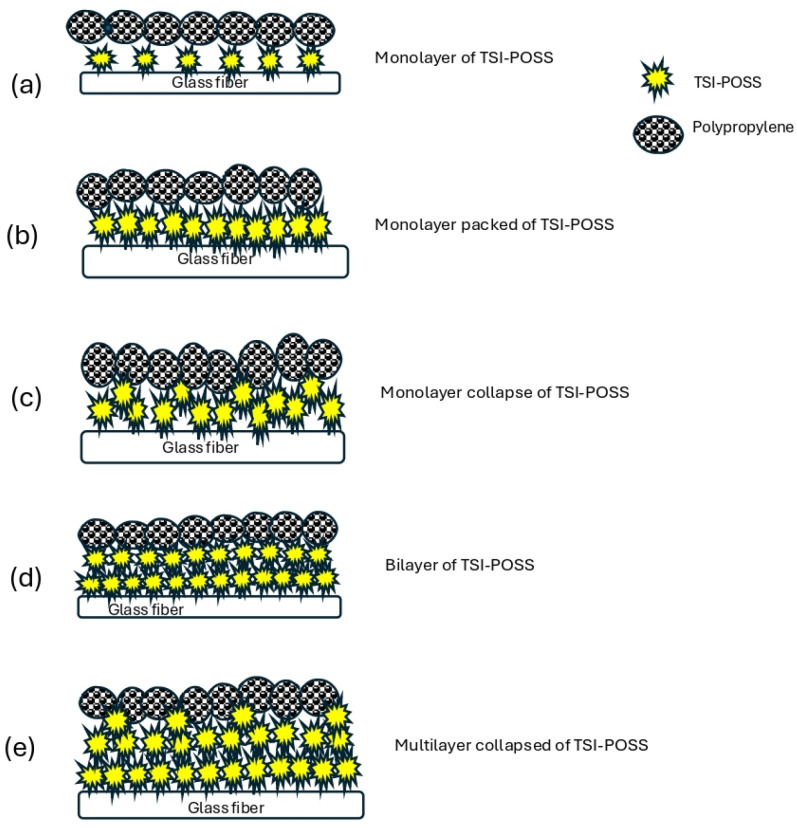
Representation of interaction suggests a TSI-POSS layer, glass fiber, and polypropylene.

The suggested representation of the TSI-POSS layers (Figure 8), polypropylene on glass fibers, according to Deng et al. [30], proposes that TSI-POSS layers are formed at the interface, in their case at the air–water interface. In the case of the interaction of TSI-POSS on glass, monolayer to multilayers of this modifier are formed. With an increasing amount of the TSI-POSS modifier, the arrangement is affected until the collapse of each layer. If we observe the behavior in the mechanical properties, it suggests that this may be due to the anti-nucleation that occurs in the composites of polypropylene with glass fibers modified with TSI-POSS, as is verified in X-rays. The increase in TSI-POSS in the glass fibers affects the decrease in the impact resistance, and the absence of β-crystals is related to the increase in TSI-POSS in the GFs in the composite.

### 3.5. Thermal Properties

**Figure 9:**
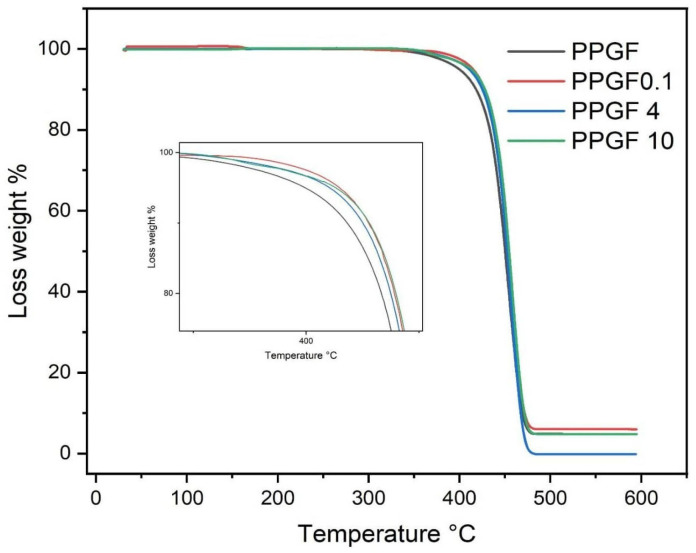
TGA analysis of PPGF, PPGF0.1, PPGF 4, and PPGF 10.

According to the thermogravimetric analysis, which was conducted under a nitrogen atmosphere, the samples with glass fiber and TSI-POSS behaved similarly to the samples with only glass fiber and polypropylene (Figure 9). The highest thermal stability of the samples with TSI-POSS was around 400 °C, while the samples containing only fibers had a thermal stability of 350 °C. At a temperature of 465 °C, it was observed that the samples without TSI-POSS and those with 0.1 and 4% TSI-POSS had the same behaviors and a residual amount of 8% polymer; however, the samples with a higher amount of TSI-POSS had a residual amount of 2% polymer [30,31,32,33,34]. Therefore, the polymer was totally consumed, and only a residual amount was present at a temperature of 465 °C, which is a clear effect of the presence of TSI-POSS. Smith et al. [35] observed the same effect of PP and POSS; the degradation temperature depended on the amount of POSS used. Other investigations using POSS with a polyethylene matrix demonstrated that POSS can improve thermal oxidative properties using polyolefin [1]. It was suggested that POSS forms a core–shell in polyolefin, and this effect can be observed in a flammability test.

### 3.6. Flammability Test

In the ASTM D635-18 test, polypropylene is given a flammability rating based on its flame spread speed, measured in millimeters per second (mm/s). If PP burns with a flame spread velocity of less than 100 mm/s, it is a low-flammability material. The material is considered flammable if the flame spread speed exceeds 100 mm/s.

In Table 8, the incorporation of TSI-POSS into the polymer with fibers is reflected in a decrease in the burning rate, decreasing from 30 mm/min to 10 mm/min in the PPGF-10 polymer. A similar effect on the burning rate was observed when using keratin fibers and chitosan composites of polypropylene as flame retardants [20,36,37]. The limiting oxygen index (LOI) value of 21% for PPGF with TSI-POSS increased by 3.5% for PP-B. The increase in the % of LOI demonstrates an increase in flame retardancy and the good compatibility of TSI-POSS/fiber/polypropylene. No significant value was obtained for the peak heat release rate (PHRR) using PPGF-POSS; this indicates an effect on flame retardancy. The PP-B sample had an ignition time of 38 s, while the PPGF-4 sample presented a total ignition time of 63 s.

## 4. Conclusions

The presence of TSI-POSS was observed with the presence of Si-OH bands in the FTIR of the glass fiber samples. In the X-ray analysis, the anti-nucleation of the polypropylene composites with the modification of the glass fibers (GF) with TSI-POSS is demonstrated; this anti-nucleation behavior causes the β-crystals to disappear. TSI-POSS had notable effects on thermal properties, which were observed to improve when increasing the amount of TSI-POSS by up to 10%. Dynamic mechanical analyses showed that the highest E″ was found in the sample with the highest amount of TSI-POSS dispersed (10%). However, according to investigations by other authors, lower amounts of TSI-POSS are related to an increase in in $T_{\alpha }^{E^{\prime \prime }}$, restricting the movement of the amorphous molecular chains of the polymer. Regarding the modification of glass fiber with TSI-POSS, the tensile strength increased with an increasing amount of TSI-POSS, and the fracture point decreased with an increasing amount of TSI-POSS. The impact strength increased to a maximum with 2% TSI-POSS and remained constant up to 10% TSI-POSS. Thermal stability slowly increased the decomposition temperature of polypropylene with increasing TSI-POSS, as observed by other authors. The flame behavior—the burn rate—decreased with increasing TSI-POSS, the % LOI was observed to increase with TSI-POSS, and the PHRR values showed no significant change. Therefore, TSI-POSS does not affect polypropylene.

## Figures and Tables

**Table 1:** Composition of the samples.

ID	Sample	% POSS Concentration	Description
Glass fiber	GFPOSS-B	-	50 g glass fiber unmodified
TSI-POSS in glass fiber	GFPOSS-0.1	0.1	50 g glass fiber + 0.05 g of TSI-POSS
TSI-POSS in glass fiber	GFPOSS-0.5	0.5	50 g glass fiber + 0.1 g of TSI-POSS
TSI-POSS in glass fiber	GFPOSS-1	1.0	50 g glass fiber + 0.5 g of TSI-POSS
TSI-POSS in glass fiber	GFPOSS-2	2.0	50 g glass fiber + 1.0 g of TSI-POSS
TSI-POSS in glass fiber	GFPOSS-3	3.0	50 g glass fiber + 1.5 g of TSI-POSS
TSI-POSS in glass fiber	GFPOSS-4	4.0	50 g glass fiber + 2.0 g of TSI-POSS
TSI-POSS in glass fiber	GFPOSS-10	10.0	50 g glass fiber + 5.0 g of TSI-POSS
		% Glass fiber	
Polypropylene	PP-B	-	PP
Glass fiber in PP	PPGF-B	-	380 g PP + 20 g GFPOSS-B
Glass fiber mod.in PP	PPGF-0.1	5.0	380 g PP + 20 g GFPOSS-0.1
Glass fiber mod.in PP	PPGF-0.5	5.0	380 g PP + 20 g GFPOSS-0.5
Glass fiber mod.in PP	PPGF-1	5.0	380 g PP + 20 g GFPOSS-1
Glass fiber mod.in PP	PPGF-2	5.0	380 g PP + 20 g GFPOSS-2
Glass fiber mod.in PP	PPGF-3	5.0	380 g PP + 20 g GFPOSS-3
Glass fiber mod.in PP	PPGF-4	5.0	380 g PP + 20 g GFPOSS-4
Glass fiber mod.in PP	PPGF-10	5.0	380 g PP + 20 g GFPOSS-5

**Table 2:** Number and size ratio of fibers analyzed.

Sample	No. of Fibers	Average Length (μm)
PPGF	18	104.44 ± 32.47
PPGF-0.1	31	30.69 ± 32.47
PPGF-0.5	25	221.98 ± 68.18
PPGF-1	40	184.43 ± 90.11
PPGF-2	40	123.09 ± 49.14
PPGF-3	39	206.29 ± 86.77
PPGF-4	22	144.63 ± 21.47
PPGF-10	35	160.86 ± 68.09

**Table 3:** Storage modulus and loss modulus values of the samples at different temperatures.

	Storage Modulus E′		Loss Modulus E″	
Sample	40 °C (MPa)	0 °C (MPa)	Max. (MPa)	$T_{\alpha }^{E^{\prime \prime }}$ (°C)
PP-B	1658.69	1404	95	13.25
PPGF-B	1784.00	1638	113.76	13.10
PPGF-0.1	1589.28	1460	137.44	8.70
PPGF-0.5	1638.67	1517	121.16	13.07
PPGF-1	1460.57	1375	125.04	9.62
PPGF-4	1876.83	1680	112.54	11.79
PPGF-10	1659.00	1489	137.93	10.26

**Table 4:** Maximum tan δ, T_g_, and T_α_* values.

	Tan δ			
Sample	Tan δ Max	T_g_ in Tan δ Max (°C)	T_α_* (°C)	Tan δ in T_α_*
PP-B	0.0833	17.73	83.26	0.1302
PPGF-B	0.0847	17.89	85.95	0.1266
PPGF-0.1	0.1165	14.59	-	-
PPGF-0.5	0.1026	18.66	89.20	0.1301
PPGF-1	0.1135	18.82	78.89	0.1268
PPGF-4	0.0820	16.55	84.64	0.1236
PPGF-10	0.1139	15.52	71.64	0.1226

**Table 5:** X-ray diffraction results for 2θ angular positions and interplanar distance “d”.

	2θ						“(110)”	“(040)”	“(130)”	“(111)”	“(131)”	“(300)”
Sample	“(110)”	“(040)”	“(130)”	“(111)”	“(131)”	“(300)”	d (Å)					
PP-B	14.24	16.98	18.68	21.27	22	16.57	6.2147	5.2175	4.7464	4.1739	4.0370	5.3457
PPGF-B	14.23	17	18.71	21.24	21.95	--	6.2190	5.2114	4.7388	4.1797	4.0461	--
PPGF-0.1	14.16	16.97	18.68	21.2	21.95	--	6.2496	5.2206	4.7464	4.1875	4.0461	--
PPGF-0.5	14.26	17.09	18.74	21.3	22.01	--	6.2060	5.1842	4.7313	4.1681	4.0352	--
PPGF-1	14.35	17.11	18.75	21.34	22.03	16.29	6.1673	5.1782	4.7288	4.1604	4.0316	5.4369
PPGF-2	14.23	17.02	18.69	21.24	21.98	--	6.2190	5.2054	4.7438	4.1797	4.0406	--
PPGF-3	14.35	17.15	18.84	21.49	22.1	--	6.1673	5.1662	4.7064	4.1317	4.0190	--
PPGF-4	14.11	16.93	18.62	21.18	21.88	--	6.2717	5.2328	4.7615	4.1914	4.0589	--
PPGF-10	14.22	17.02	18.68	21.27	21.95	--	6.2234	5.2054	4.7464	4.1739	4.0461	--

**Table 6:** X-ray diffraction results for response intensity and crystal size.

	Intensity						“(110)”	“(040)”	“(130)”	“(111)”	“(131)”	“(300)”
Sample	“(110)”	“(040)”	“(130)”	“(111)”	“(131)”	“(300)”	Size of Crystal “D” (Å)					
PP-B	9891	7727	3537	2678	3843	974	159.02	211.52	172.76	150.41	129.99	10.68
PPGF-B	3224	6127	1657	1105	1119	--	136.89	194.00	122.02	58.89	201.62	--
PPGF-0.1	2698	4138	1250	858	715	--	124.48	174.50	100.12	54.35	204.87	--
PPGF-0.5	6504	6153	2891	2461	3345	--	148.85	195.20	147.93	146.48	150.65	--
PPGF-1	5976	9685	2415	1439	2222	757	167.99	206.17	169.32	146.24	133.28	15.63
PPGF-2	6286	5989	2826	2388	3189	--	139.82	180.31	110.94	114.28	146.89	--
PPGF-3	4076	2765	1272	2550	379	--	100.50	157.34	21.96	63.10	43.35	--
PPGF-4	3543	5223	1641	1177	1536	--	151.61	201.24	151.03	146.98	145.22	--
PPGF-10	6659	6197	2753	2091	2891	--	151.58	190.86	136.93	123.55	137.92	--

**Table 7:** FHWM results for each representative peak per sample.

	FWHM (rad)					
Sample	“(110)”	“(040)”	“(130)”	“(111)”	“(131)”	“(300)”
PP-B	0.00918	0.00692	0.00849	0.00980	0.01135	0.13692
PPGF-B	0.01066	0.00755	0.01203	0.02502	0.00732	--
PPGF-0.1	0.01173	0.00839	0.01466	0.02711	0.00720	--
PPGF-0.5	0.00980	0.00750	0.00992	0.01006	0.00979	--
PPGF-1	0.00869	0.00710	0.00867	0.01008	0.01107	0.09360
PPGF-2	0.01044	0.00812	0.01323	0.01289	0.01004	--
PPGF-3	0.01452	0.00931	0.06683	0.02336	0.03403	--
PPGF-4	0.00962	0.00728	0.00972	0.01002	0.01016	--
PPGF-10	0.00963	0.00767	0.01072	0.01193	0.01070	--

**Table 8:** Flame behavior of copolymers, burning rate, LOI, PHRR, THR, TTI data.

	Burning Rate (mm/min)	LOI (%)	PHRR KW/m^2^	THR MJ/m^2^	TTI (S)
PP-B	30	17.5	2000	90	38.0
PPGF-0.1	19.9	21	1429.9	91.6	35.0
PPGF-0.5	10.9	21	1292.7	170	38.6
PPGF-1	15.0	21	1446.4	165	36.5
PPGF-4	15.0	21	1504.5	165	63.0
PPGF-10	10.0	21	1215.3	170	37.5

## Data Availability

The original contributions presented in the study are included in the article, further inquiries can be directed to the corresponding author.
